# The functional mechanism of bone marrow-derived mesenchymal stem cells in the treatment of animal models with Alzheimer’s disease: crosstalk between autophagy and apoptosis

**DOI:** 10.1186/s13287-022-02765-8

**Published:** 2022-03-03

**Authors:** Chuan Qin, Lin Bai, Yongning Li, Kewei Wang

**Affiliations:** 1 Institute of Laboratory Animal Sciences, Chinese Academy of Medical Sciences and Comparative Medical Center, Peking Union Medical College, 5 Panjiayuan Nanli St., Beijing, 100021 China; 2 Department of International Medical Service and Department of Neurosurgery, Peking Union Medical College Hospital, Chinese Academy of Medical Sciences and Peking Union Medical College, Shuaifuyuan 1, Dong Cheng District, Beijing, 100730 China

**Keywords:** Alzheimer’s disease, Bone marrow-derived mesenchymal stem cells, Autophagy, Apoptosis

## Abstract

The transplantation of bone marrow-derived mesenchymal stem cells (BMMSCs) alleviates neuropathology and improves cognitive deficits in animal models with Alzheimer’s disease. However, the underlying mechanism remains undefined. Based on meta-analysis and comprehensive review, high-profile studies support the theory that transplanted BMMSCs activate autophagy, as evidenced by the expression levels of signal molecules such as Beclin-1, Atg5, LC3-II, and mTOR. Functional autophagy mitigates neuronal apoptosis, which is reflected by the alterations of IAPs, Bcl-2, caspase-3, and so forth. Moreover, the transplantation of BMMSCs can decrease aberrant amyloid-beta peptides as well as tau aggregates, inhibit neuroinflammation, and stimulate synaptogenesis. There is a signal crosstalk between autophagy and apoptosis, which may be regulated to produce synergistic effect on the preconditioning of stem cells. Forasmuch, the therapeutic effect of transplanted BMMSCs can be enhanced by autophagy and/or apoptosis modulators.

## Introduction

Alzheimer’s disease (AD) is characterized by the accumulation of aberrant Aβ peptide plaques and neurofibrillary tau tangles in pathology [1]. The pathogenesis of Alzheimer’s disease is regulated by the signal crosstalk between autophagy and apoptosis [2–4]. The transplantation of BMMSCs activates autophagy and inhibits apoptosis, improving memory and cognitive deficits in animal models with Alzheimer’s disease [5]. Herein, the relevant mechanisms are recapitulated based on the existing literature and experimental evidence.

## Alzheimer’s disease and drug treatment

**Fig. 1:**
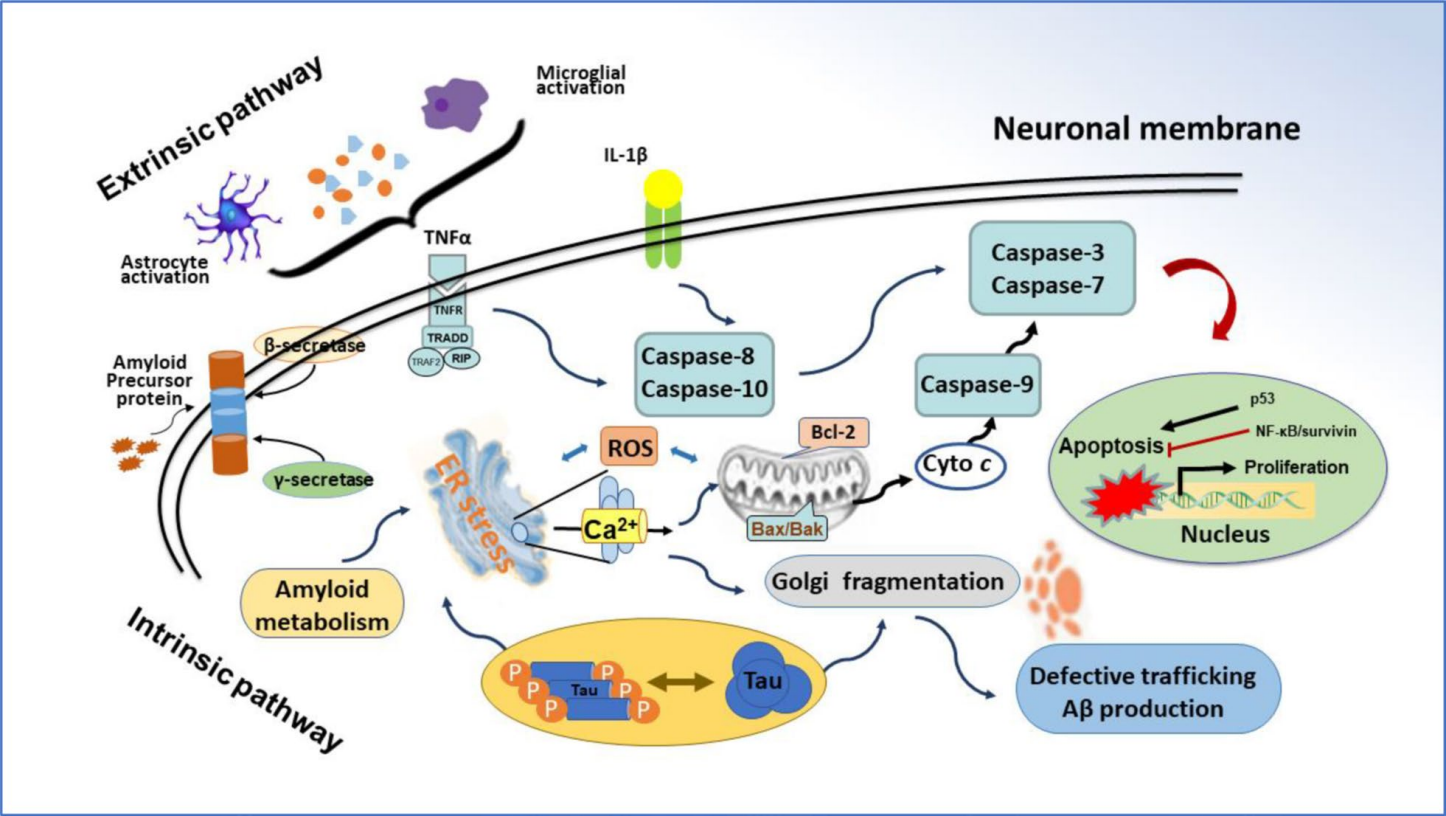
Alzheimer’s disease and neuronal apoptosis. Apoptosis is conducted through intrinsic and extrinsic pathways in the pathogenesis of Alzheimer’s disease. Inflammatory cytokines, such as TNFα, IL-6 and IL-1β, can trigger the neuronal apoptosis through membrane receptor or extrinsic pathway (i.e., TNFα/caspase-8/caspase-3). The intrinsic pathway of neuronal apoptosis is activated by intracellular insults including tau aggregates, mitochondrial dysfunction, endoplasmic reticulum (ER) stress, free radicals, etc. Mitochondrial cytochrome *c* (Cyt *c*) is released to initiate apoptosis signaling through the Cyto *c*/caspase-9/caspase-3 cascade. The pathological manifestations of Alzheimer’s disease depend on the comprehensive effect of diverse mechanisms. There is a complicated network to modulate the development of Alzheimer’s disease. The red line represents inhibitory effect. Bcl-2, B-cell lymphoma 2; IL-1β, interleukin-1β; ROS, reactive oxygen species

AD is a neurodegenerative disorder as showed by memory loss and cognitive impairment in clinical manifestations [1]. The pathological characteristics of AD are exhibited by the extracellular plaques of amyloid beta (Aβ) peptides and the hyper-phosphorylation of tau protein in neurofibrillary tangles [6]. Apoptotic cell death is induced by aberrant Aβ peptides and tau aggregates in the hippocampus and temporal lobe. Neuronal apoptosis can be carried out through intrinsic and extrinsic pathways in the pathogenesis of AD [7]. Cerebral Aβ deposits can activate microglia to release inflammatory cytokines such as TNF-α, IL-6 and IL-1β, which induce neuronal apoptosis through membrane receptors or the extrinsic pathway [8]. Hyper-phosphorylated tau aggregates may disturb intracellular homeostasis, leading to endoplasmic reticulum (ER) stress, ROS generation, oxidative stress, and abnormal energy metabolism [7]. Intracellular insults can initiate neuronal apoptosis via mitochondrial dysfunction or intrinsic pathway (Fig. 1). The pathophysiological characteristics of AD depend on the location and severity of neuropathology, which is derived from the comprehensive effect of different mechanisms such as autophagy, oxidative stress, apoptosis, inflammation, and immunoregulation. The above-mentioned mechanistic links are comprised in a complicated signal network that modulates the development of AD. The clinical treatment of Alzheimer's disease remains a challenge since the pathogenesis is not fully understood. Nowadays, there is no cure for Alzheimer's disease. Most clinical treatments are symptom-specific or exploratory. Current medicines include acetylcholinesterase (AChE) inhibitors (i.e., donepezil, galantamine and rivastigmine), NMDA receptor antagonists (i.e., memantine), and Aβ-directed monoclonal antibody aducanumab [9]. These drugs only show modest benefits for certain patients. In addition, some natural compounds have beneficial effects, such as ginsenosides, curcumin, and flavonoids. Ginsenosides could decrease Aβ_1-42_-induced neurotoxicity and tau-hyperphosphorylation [10]. Ginsenosides Rg1 and Rb1 belong to AChE inhibitors as well. Curcumin reduced the cerebral accumulation of Aβ peptides and protected neurons from the attack of free radicals [11]. Flavonoids significantly improved cognitive impairment through a variety of mechanisms such as the inhibition of cholinesterase, free radical scavenging, the modulation of signal pathways ERK and PI3K/Akt, and the suppression of apoptosis [12]. Also, non-drug therapies can help improve Alzheimer’s disease, including health diet, regular exercise, and special care.

## An overview of stem cell therapy for Alzheimer’s disease

Stem cell therapy has been explored in the preclinical study using animal models with Alzheimer’s disease [13]. The sources of stem cells may be autologous, allogenic, or iPS-derived. Generally, allogenic stem cells are prepared from placenta, umbilical cord, and embryonic tissue [14–16]. Autologous stem cells are isolated from brain, fat, or bone marrow [17–19]. There are two major problems associated with allogeneic stem cells, including ethical issue and allogeneic immunogenicity. These problems are difficult to be solved in the short term [20]. Thereupon, autologous stem cells are preferred [21]. Available research data have demonstrated that autologous stem cells from brain, bone marrow or fat have beneficial effect in alleviating the neuropathology of animal models with Alzheimer’s disease. In practice, autologous stem cells from patient’s brain tissue will front onto unacceptable attitude and technical challenges. Previous studies have also compared the therapeutic efficiency of autologous stem cells from bone marrow with that of adipose stem cells. Interestingly, the therapeutic effect of stem cells from bone marrow was better than that from the adipose tissue [8, 22, 23]. Even a single transplantation of the BMMSCs could obtain a positive result [24–26]. In addition, the delivery methods of stem cells can influence therapeutic effect. Current approaches include intravenous, intracerebral, and intracerebroventricular [5]. It appears that all these procedures are practicable, but their therapeutic advantages need to be determined through parallel comparative studies in the future. The rationality of transplanted BMMSCs is based on the following points: (a) clinical feasibility in the future. The transplantation of autologous stem cells does not involve potential issues such as ethical issue or immune-mediated adverse events; (b) therapeutic effect. The emerging evidence demonstrates that the therapeutic effect of BMMSCs is better than that of adipose stem cells [8, 23]; (c) stem cell preparation. Numerous BMMSCs can be acquired through single aspiration. Autologous bone marrow provides enough stem cells, not requiring in vitro expansion; (d) technical hassle. Although there is a little difficulty in extracting bone marrow from elderly patients, clinical experience has showed that this problem can be solved through technical improvement. State-of-the-art technology guarantees the clinical application of autologous BMMSCs.

## The transplanted BMMSCs activate autophagy

### Autophagy and Alzheimer’s disease

Autophagy sequestrates cytoplasmic components into autophagosome for subsequent degradation and recycling. Functional autophagy participates in the removal of Aβ peptide as well as the assemblance of tau protein in cerebral tissue [1, 27, 28]. In the pathological region of Alzheimer’s disease, aberrant Aβ peptides are accumulated. The dysregulation of autophagy exacerbates the progression of AD [29, 30]. In contrast, an appropriate activation of autophagy alleviates neuropathology as revealed by the expression levels of molecular markers such as Beclin-1, atg7, LC3, Lamp-1, Lamp-2, and mTOR [27, 31, 32]. Among them, the core component of mTOR complex is associated with the elimination of Aβ proteins by regulating the key signal pathways PI3K/Akt, GSK-3, AMPK, and IGF-1 [33–35]. The stimulation of mTOR contributes to the intracellular level of hyperphosphorylated tau protein [35]. The autophagy dysfunction in the early stage of AD is manifested by abnormal mitophagy and subsequent aberrant Aβ and tau pathology [36]. The decreased mitophagy is connected with synaptic decline and cognitive deficits as evidenced in animal models as well as in patients with sporadic late-onset AD [37]. The malfunction of autophagy may take place at any stage of the multi-step process. After the fusion of autophagosome with lysosome, the adequate activity of lysosomal enzymes eliminates the intracellular burdens of aggregated proteins and oxidized lipids, which relieves stressful insults and the buildup of ROS [38]. Instead, aging or genetic factor can dwindle the degradability of lysosomal enzymes, leading to the accumulation of aberrant Aβ peptides and tau aggregates. The deficiency of lysosomal enzymes (e.g., NEU1) affects exocytosis and Aβ secretion [38]. Damaged lysosomes involve peroxide gathering and the spreading of tau protein [39]. Oxidative injury destroys membrane integrity, causing aggregates to be released into the cytosol. Cytosolic aggregates may act as seeds for further scale-up, leading to apoptosis [40]. Lysosome biogenesis can be boosted to rescue tau-mediated neurotoxicity [41–43].

### Transplanted BMMSCs and functional autophagy

After the transplantation of BMMSCs, behavioral and cognitive impairments are improved in AD-like models as demonstrated by Morris water maze test, Y-maze alternation test, plus-maze discriminative avoidance task, social recognition test, and open-field evaluation [5, 44, 45]. In neuropathology, the transplanted BMMSCs can alleviate the levels of aberrant Aβ and hyperphosphorylated tau proteins, which abate neuronal apoptosis. Moreover, the reduced levels of Aβ plaques and tau phosphorylation are beneficial in both young and aged AD-like animals [46, 47]. All of cognitive and pathological changes are related to the enhancement of autophagy [8, 18]. The transfused MSCs can upregulate the expression of BECN1/Beclin 1 and increase LC3-II-positive autophagosomes in the hippocampus, which stimulate the clearance of Aβ peptides in AD-like models [29]. Also, the activation of autophagy relieves nerve injury through the mitigation of oxidative stress [29]. Following the transplantation of BMMSCs, the activated autophagy can promote the gene expression of cell growth. Cellular proliferation is modulated by PI3K, Akt and ERK1/2 signaling pathways [48, 49]. The therapeutic effect of BMMSCs is not only verified by direct transfusion into the cerebral area, but also by the peripheral blood mobilization of bone marrow stem cells after the injection of macrophage colony-stimulating factor (M-CSF) [50]. Motivated stem cells in the blood stream can migrate into the pathological area of cerebral parenchyma in the APP/PS1 mouse [50].

### Autocrine and paracrine cytokines

**Fig. 2:**
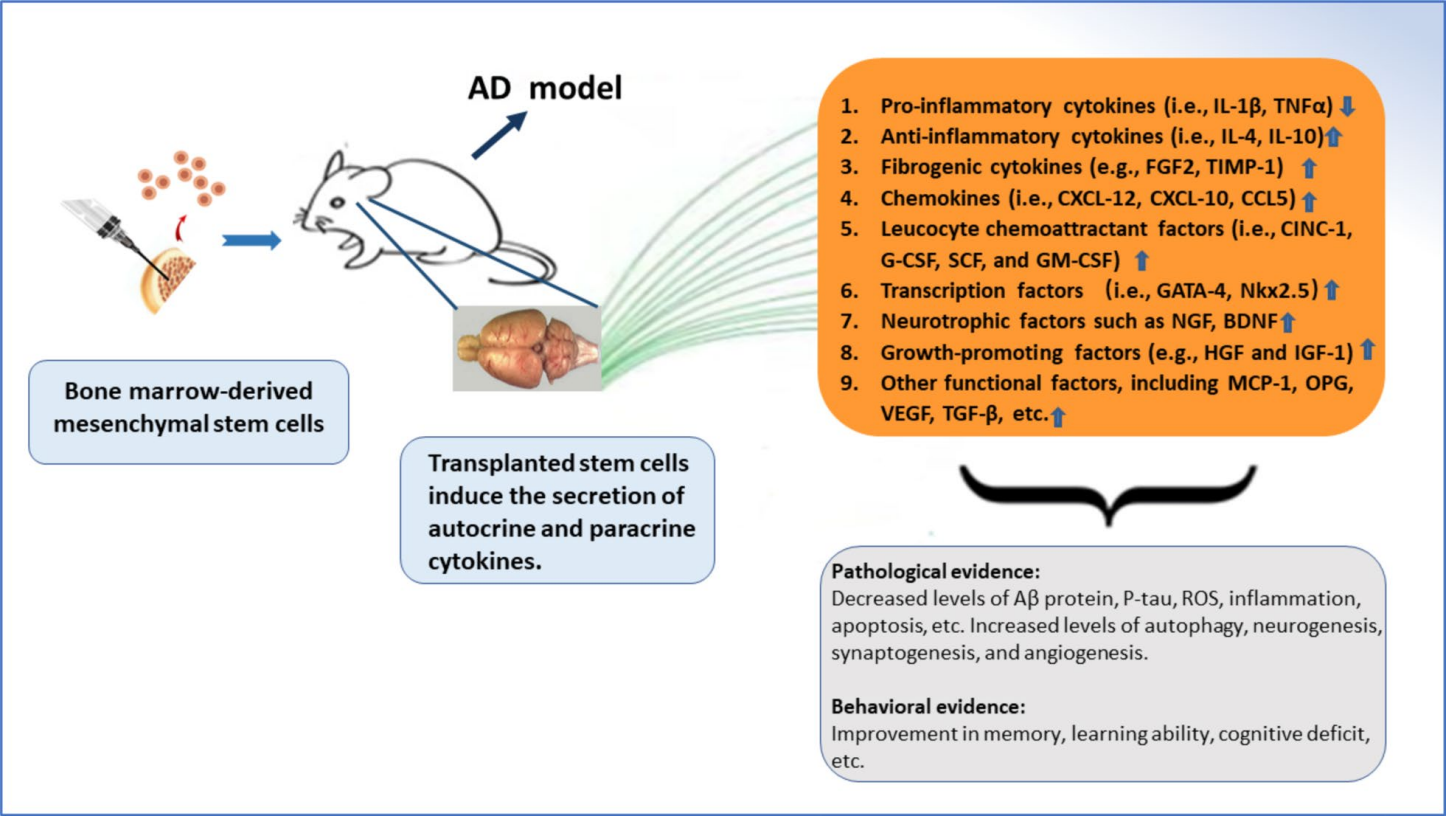
Secretion of autocrine and paracrine cytokines is induced by the transplanted BMMSCs. These factors have diverse functions. For instance, IL-4 and IL-10 can suppress inflammatory role and exert positive effect. GM-CSF recruits peripheral monocytes into the lesion. These monocytes are further activated by extracellular Aβ proteins, which accelerate Aβ clearance in APP/PS1 mice. TGF-β participates in multiple signaling pathways to mediate amyloid metabolism, immunoregulation, and neuroprotection. The comprehensive effect of functional autocrine and paracrine cytokines determines the therapeutical potential of BMMSC

The transplanted BMMSCs have the characteristics of self-renewal, proliferation, and differentiation into tissue-specific cell lineages. The transplantation of BMMSCs alters local microenvironment and produces a variety of autocrine and paracrine cytokines (Fig. 2), including (i) inflammatory cytokines (i.e., IL-1, IL-6, IL-10 and TNFα); (ii) fibrogenic cytokines (e.g., FGF2, TGF-β, TIMP-1) [51–53]; (iii) chemokines (CXCL-12, CXCL-10, CCL5, etc.) [52, 54, 55]; (iv) leucocyte chemoattractant factors (CINC-1, G-CSF, SCF, GM-CSF and so forth) [56]; (v) transcription factors such as GATA-4, Nkx2.5 and MEF2C [57]; (vi) neurotrophic factors and growth-promoting factors such as NGF, BDNF, HGF, and IGF-1 [54, 58–61]; (vii) other functional factors, including MCP-1, OPG and so on [8, 54, 62]. Certain cytokines are common products that can be generated by different types of stem cells, whereas other cytokines such as CXCL-12 and SDF-1 are only secreted by BMMSCs [63]. These autocrine and paracrine cytokines are regulated by various factors such as (a) age. The level of IL-6 in human bone marrow is positively correlated with age. The secretion of immunoreactive IL-6 and IL-11 is also increased with age [64]; (b) gender. Women receiving estrogen replacement therapy show a low secretion of IL-6 and IL-11 [64, 65]; (c) local conditions. Paracrine cytokines are influenced by regional blood supply, the interaction between stem cells and glial cells, delivery methods and so on [52, 54, 66]. Preconditioning or modified MSCs can elevate the efficiency of stem cell therapy in neurodegenerative disease [18, 67, 68].

### Signaling pathways related to autophagy activation

**Fig. 3:**
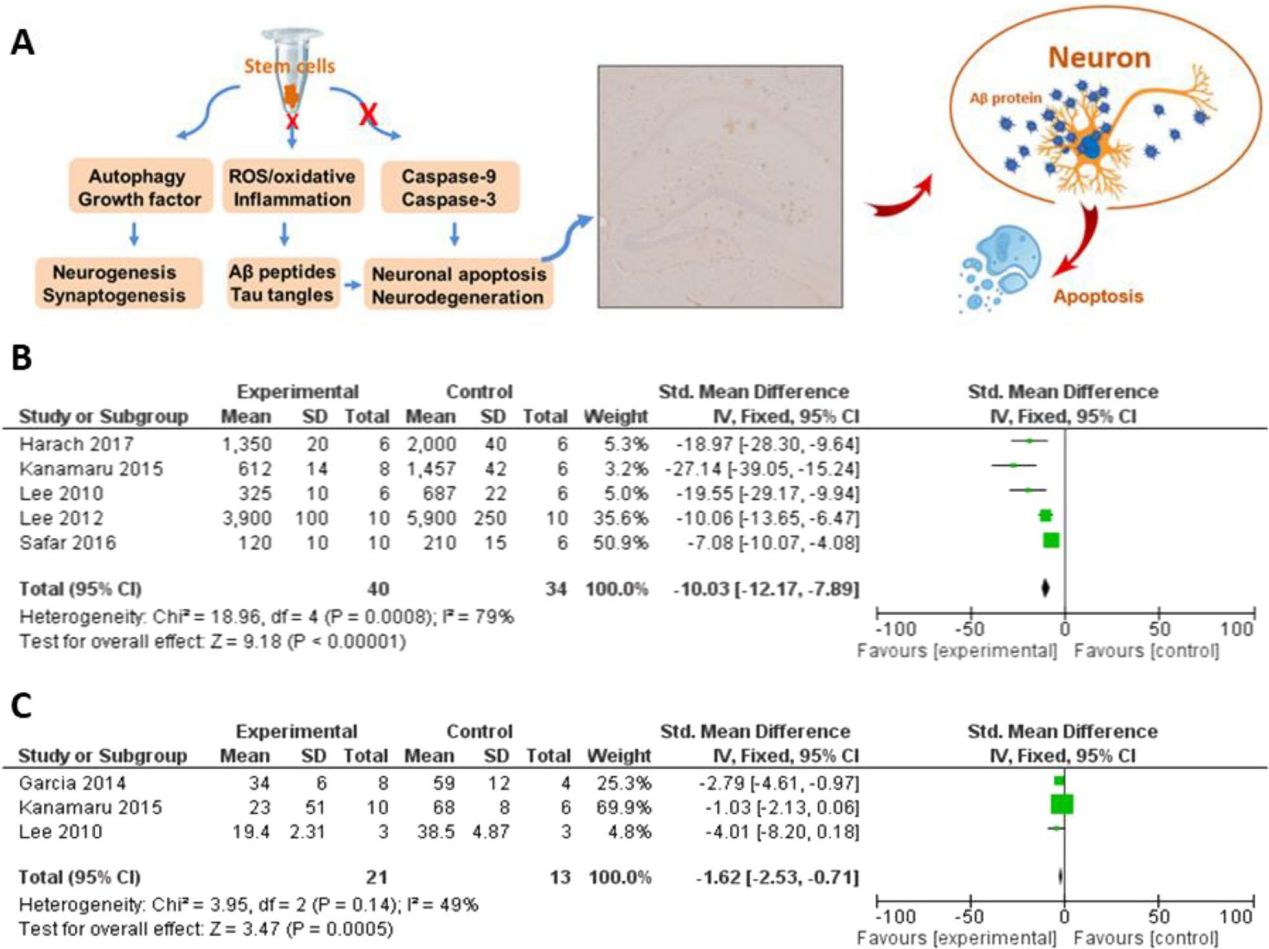
Apoptosis plays an important role in the pathogenesis of Alzheimer’s disease. Aberrant Aβ plaques were accumulated in the hippocampus of APP/PS1 mice, which induced neuronal apoptosis (**A**). The meta-analysis demonstrated that the transplantation of BMMSCs could decrease the level of soluble Aβ proteins (**B**) and inhibited the activation of caspase-3 (**C**)

Genetic modification indicates that the down-regulation of Becn-1 increases intraneuronal Aβ production and extracellular Aβ deposition, whereas the enhancement of Beclin-1 expression decreases amyloid pathology in APP transgenic mice [69]. The expression of Beclin-1 is negatively correlated with the level of Aβ proteins, which provides a mechanistic link between activated autophagy and the inhibition of apoptosis (Fig. 3). Beclin-1 can directly bind to antiapoptotic Bcl-2 proteins such as Bcl-2, Bcl-xL, Bcl-w and Mcl-1 [70]. The induction of autophagy is initiated, while Beclin-1 is dissociated at BH3-only domain from Bcl-2 proteins due to the phosphorylation of Bcl-2 by JNK or competitive combination with other pro-apoptotic Bcl-2 protein (e.g., Bad). Autophagic response can be balanced by the caspase activation. Activated caspase-8 cleaves Beclin-1 into C-terminal and N-terminal fragments to trigger apoptosis [71]. Cell fate is modified by caspase activity and the interaction of diverse BH3 proteins with Beclin-1 [70]. The transplantation of BMMSCs alters zonal microenvironment through the secretion of autocrine and paracrine cytokines. The beneficial effect of BMMSCs may be through the upregulation of BECN1/Beclin-1 expression, the modulation of Bcl-2 family, and the inhibition of caspases [29, 72]. The interaction between Beclin-1 and Bcl-2 proteins takes part in the PI3K class III pathway [73, 74]. The expression of Seladin-1 and nestin is also associated with the PI3K/AKT and ERK1/2 signaling pathways in the hippocampus [48]. PI3K-AKT signaling plays an important role in synaptic plasticity as well as intracranial brain volume, which is also required for cell growth and apoptosis [75]. The PI3K/AKT/mTOR pathway regulates cell cycle, which is necessary to promote the proliferation of neural progenitor cells in adult hippocampus [76]. The inhibition of mTOR (e.g., energy depletion, starvation, or hypoxia) stimulates activation autophagy and energy metabolism [77]. So far, certain mammalian modulators targeting mTOR-dependent or independent autophagy have been identified, which show positive effects in the treatment of AD [33]. These considerable data support the pathophysiological significance of autophagy in the pathogenesis of AD.

### The different responses of various cell types

After BMMSCs are transplanted into cerebral tissue, the responses from different cell types such as neurons, astrocytes, and microglia are varied. In neurons, the transplanted BMMSCs stimulate the activation of autophagy, which promotes (a) neurogenesis, differentiation, and integration; (b) synaptic plasticity; and (c) the inhibition of apoptosis. The transplanted BMMSCs mediate immunomodulation and relieve neuroinflammation derived from astrocytes [78]. In vitro study revealed that the expression of pro-inflammatory factors such as IL-1β, TNFα, and IL-6 in astrocytes was attenuated following exposure to MSC-conditioned medium [49, 78]. Furthermore, cytokines released by MSCs could favor cell proliferation through the regulation of intermediate filaments (GFAP, vimentin), pro-inflammatory enzymes (iNOS, COX-2) and receptors (TLR4, CD14, mGluR3, mGluR5) [78]. The transplantation of BMMSCs activates quiescent microglia and recruits peripheral monocytes into the lesion [79]. Microglial activation has two consequences, including (i) beneficial effects such as the clearance of Aβ peptides and the mitigation of tauopathy; (ii) adverse effects. Activated microglia may release inflammatory cytokines to trigger neuronal apoptosis through membrane receptor pathway. Accordingly, the activation of microglia is a two-edged sword in cerebral tissue. The beneficial or harmful effects depend on the integrative result of multiple signal crosstalk. Nevertheless, available data demonstrate that transplanted BMMSCs participate in the regulation of immune and inflammatory responses. The autophagy mechanism by which the transplantation of BMMSCs alleviates neuropathology and ameliorates the cognitive function of AD-like animals may involve signal molecules such as Beclin-1, atg7, LC3, Lamp-1 and Lamp-2, and mTOR [80, 81]. The comprehensive effect of transplanted BMMSCs benefits the improvement of Alzheimer’s disease.

Functional microglia play a pivotal role in neuroinflammation and immunomodulation. The transplanted BMMSCs inhibit microglial activity to alleviate neuropathology in different AD-like models [78]. Particularly, the transplantation of stem cells can shift microglial phenotype M1 toward M2. M1/M2 polarization attenuates the secretion of pro-inflammatory cytokines in M1 microglia, but induces the production of anti-inflammatory cytokines in M2 microglia [82–84]. Obviously, the transplantation of BMMSCs initiates immunoregulatory mechanisms, including peripheral monocyte recruitment, microglial M1/M2 polarization, dramatic reversal in pro-/anti-inflammatory cytokine profile, neurotraphin-mediated synaptic plasticity, and so on [5, 18, 85]. In general, inflammation/ immunoregulation is key axis associated with the improvement of synaptic function and cognitive performance, which can be shaped by the crosstalk between autophagy and apoptosis pathways [86–88].

### Stem cell therapy may be enhanced by drug treatment

**Fig. 4:**
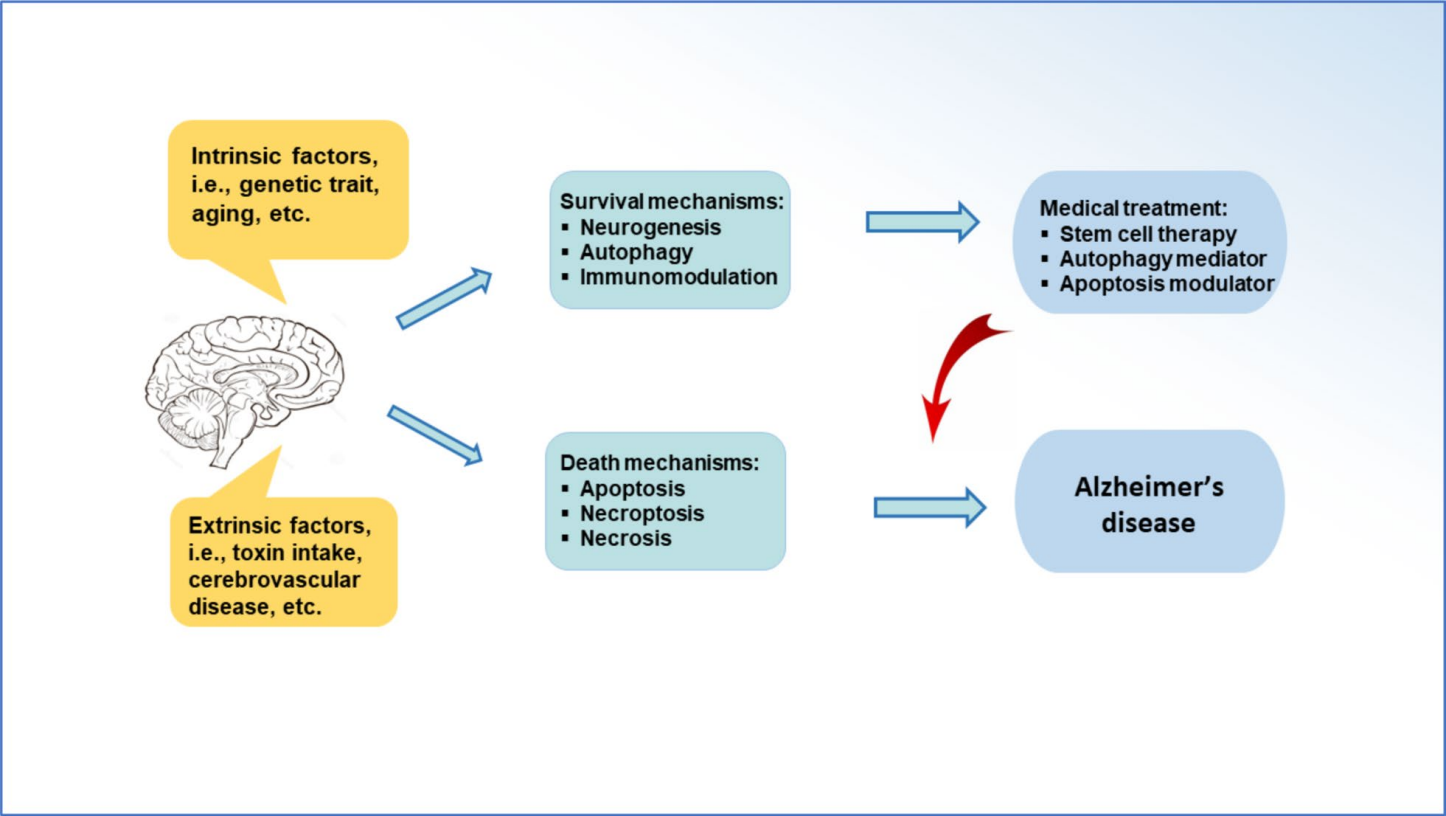
Synergistic effect of stem cell therapy with autophagy activation. The transplantation of BMMSCs stimulates autophagy and inhibit apoptosis, which improves memory and cognitive function in animal models with Alzheimer’s disease. When the transplanted BMMSCs is combined with drug regulators, it is hypothesized that a better therapeutic effect can be acquired

Presently, drug development for AD treatment is based on a limited understanding of related mechanisms, such as the clearance of Aβ peptides, the removal of tau aggregates, the inhibition of apoptosis and oxidative stress, and the activation of autophagy. In particular, the central role of autophagy in the progression of AD has been confirmed by a large amount of evidence [30, 89]. Dysfunctional autophagy impedes neuronal survival and causes apoptotic cell death. The appropriate regulation of autophagy is a potential target to block AD development. For instance, the stimulation of mitophagy may prevent the neurodegeneration in AD [37]. The certain regulators of mTOR-dependent and independent autophagy have showed beneficial effects in the improvement of AD [28, 90]. The functional MSCs can enhance LC3-II expression and the number of LC3-II-positive autophagosomes [29, 91]. When MSCs are administrated into AD-like animal models, the enhancement of autophagy activity reduces the level of hippocampal Aβ peptides and facilitates neuronal survival following the upregulation of BECN1/Beclin-1 expression (29). The modulation of autophagy by chemical drugs, miRNAs, or cytokines will be a plausible method to improve the efficiency of therapeutic MSCs [24, 26, 29]. Possibly, there is a synergistic effect if the transplanted BMMSCs is combined with autophagy mediators (Fig. 4).

## Mechanism of BMMSCs-inhibiting apoptosis

### The transplanted BMMSCs inhibit apoptosis

Apoptosis causes neuronal death and memory loss in AD-like animals, which can be reversed by the transplantation of BMMSCs. Apoptosis mechanism participates in not only the pathogenesis of AD, but also the survival of transplanted BMMSCs in the lesion [68]. Apoptosis signaling pathway can be regulated at different levels, including (a) the activation of nuclear factors such as p53, Foxa2, C/EBPβ and so on; (b) the enhancement of anti-apoptotic proteins such as Bcl-2, survivin, XIAP; and (c) the indirect regulation of signal molecules SDF-1, NGF, etc.

1. Direct effects
  - Inhibition of caspases. The transplanted BMMSCs attenuate apoptotic cell death in the AD-like mice by inhibiting caspase-3 activation as discovered by immunohistochemical staining and quantitative image analysis [59, 92, 93]. Potential mechanisms may be related to the inhibition of caspase-3 activities by anti-apoptotic Bcl-2 [72]. Also, the transplanted BMMSCs can increase the number of positive cells expressing survivin and seladin-1 [94, 95]. Survivin interacts with caspases to avoid its cleavage and activation, resulting in the blockage of the apoptotic cascade [96]. The neuroprotective seladin-1 prevents the activation of caspase-3 in the AD groups [97].
  - IAPs family. The transplantation of BMMSCs enhances the number of survivin-positive cells in AD models [94, 95]. Anti-apoptotic survivin inhibits the activation of intracellular caspases and disrupts the signal pathway of apoptosis. The over-expression of XIAP in BMMSCs can suppress neuronal apoptosis in rats with cerebral palsy [98]. Beclin-1-dependent autophagy is induced by the amplification of XIAP and cIAP1 [99].
2. Indirect effects
  - Elimination of Aβ peptides. The accumulation of Aβ deposits is a typical marker related to neuronal loss. Aberrant Aβ peptides are able to induce apoptosis through regulators such as stress-activated protein kinases p38, c-Jun N-terminal kinase, and p53 expression [1]. Aβ peptides-caused neuronal apoptosis in the AD-like animals is conducted via classic caspase activation. Stem cells can diminish Aβ-mediated apoptosis in co-cultured hippocampal neurons as well as in AD-like animals derived from Aβ-intrahippocampal injection [72, 100].
  - Apoptosis inducing factor (AIF). Apoptosis can be induced through caspase-independent pathway as well [101, 102]. Mitochondrial AIF is transferred into the nucleus to recruit nucleases and induce apoptosis as exhibited by chromatin condensation and DNA fragmentation. AIF could be embedded in neurofibrillary tangles as confirmed in postmortem study using neuron immunoreactivity [102, 103]. There was a significant increase in AIF expression in the hippocampus and temporal cortices, showing the positive correlation between nuclear AIF-positive number and Braak stages in AD samples [101]. The AIF-induced neuronal apoptosis in the early stage of AD could be observed in the hippocampus, amygdala, and basal forebrain [102]. At present, no research result shows direct evidence that the transplanted BMMSCs have an inhibitory effect on the AIF.
  - Activation of nuclear factors. Apoptosis is linked to the activation of nuclear factors such as p53, NF-κB, C/EBPβ, and Foxa2 [104–106]. For instance, p53 induces autophagy in a DRAM-dependent manner [105]. DRAM is an effector of p53-mediated apoptosis. BMMSCs can alleviate apoptosis in the hippocampus to exert neuroprotective role, which may be related to p53-mediated senescence [100, 107]. The transplanted BMMSCs also decrease the levels of p53 and p21 in the aging cells [108].
  - Oxidative stress. The pathogenesis of Alzheimer’s disease is associated with oxidative stress as reflected by glutathione level, ROS production, peroxidation, and the activities of oxidation-related enzymes, which involves ER stress and mitochondrial dysfunction [89, 109]. The transplantation of MSCs can stimulate mitophagy to eliminate oxidized components and aberrant proteins. The transfusion of MSCs may increase the comprehensive capability of the endogenous antioxidant system to neutralize oxidative stress [49].
  - Other effects. MSCs produce autocrine and paracrine cytokines, among which VEGF promotes angiogenesis and neurogenesis [49, 110]. Seladin-1, a key modulator of apoptosis, inhibits the activation of caspase-3 [111]. The transplanted BMMSCs significantly protected seladin-1 from cleavage [48]. The beneficial effects also came from neurotrophic factors such as NGF, FGF2, and BDNF [60, 61].

### Interaction between autophagy and apoptosis

**Fig. 5:**
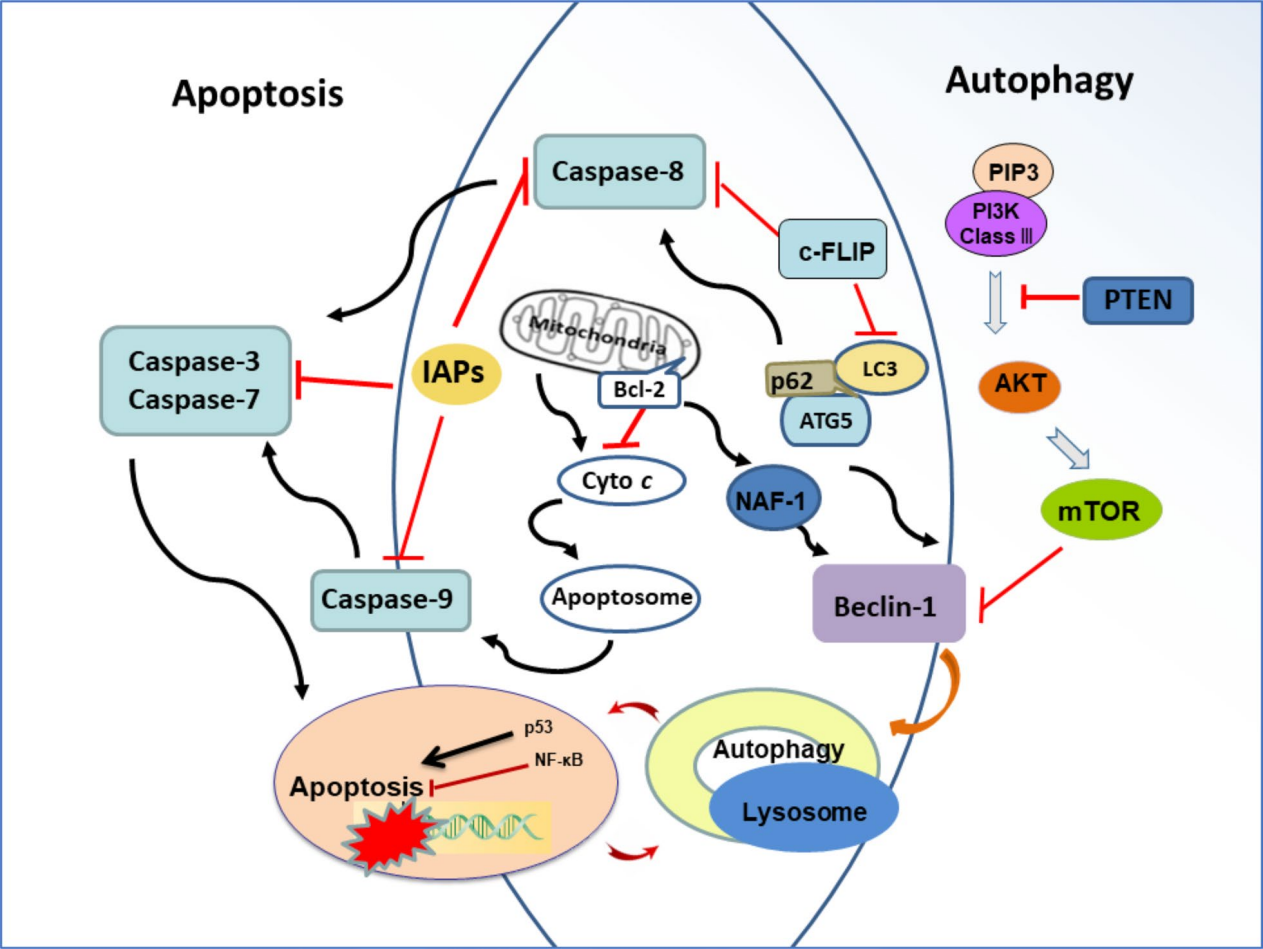
Interaction between autophagy and apoptosis. The downregulation of autophagy causes neural apoptosis. Autophagy is able to accelerate apoptosis via the degradation of IAPs as well. Apoptosis inhibits autophagy in enzyme-dependent manners. There is signal crosstalk between apoptosis and autophagy by sharing common regulators such as p53, Atg5, caspase-8, Beclin-1/Bcl-2, and IAPs. IAPs, inhibitors of apoptosis proteins; Bcl-2, B-cell lymphoma 2; Cyto *c*, cytochrome *c*; c-FLIP, cellular FLICE-like inhibitory protein; PIP3, phosphatidylinositol 3,4,5-trisphosphate; PI3K, phosphatidylinositol 3-kinase; PTEN, phosphatase and tensin homolog; AKT or PKB, protein kinase B; mTOR, mammalian target of rapamycin; ATG5, autophagy related 5; LC3, microtubule-associated proteins 1A/1B light chain 3B; p53, tumor protein P53; NF-κB, nuclear factor kappa-light-chain-enhancer of activated B cells

The pathogenesis of Alzheimer’s disease is regulated by the interaction between autophagy and apoptosis (Fig. 5). Decreased autophagy is accompanied by neural apoptosis [2, 29, 89]. There is a complicated network composed of signaling molecules such as mTOR, Beclin1, and HSPB1, which modulates the interaction between autophagy and apoptosis in the cerebral tissue [35]. The crosstalk between apoptosis and autophagy shares common regulators such as p53, Atg5, caspase-8, Beclin-1/Bcl-2, and IAPs [93, 105, 112–114]. Bcl-2 protein may affect signal molecules such as Beclin1 and Bcl-xL [112]. cFLIP mediates LC3 conjugation and inhibits the activation of caspase-8 [114]. The complex consisting of Atg5, LC3, and p62 modulates autophagosome formation and caspase-8 activation [115, 116]. Autophagy is associated with apoptosis through the connection of lysosomal damage/mitochondrial dysfunction. Normal lysosome grants the functional performance of autophagy. Lysosomal damage induced by abnormal aggregates leads to seed-like propagation and subsequent apoptotic cell death via mitochondrial pathway [29, 69, 103, 117, 118]. Drug intervention can modulate the interaction between autophagy and apoptosis, which may be a plausible strategy for the clearance of aberrant proteins and thus delay the onset of AD. Accordingly, the appropriate regulation of autophagy is a practicable target for the development of therapeutic drugs.

## Challenges and perspective

### Uncertainty

Although numerous data support the beneficial role of autophagy activation in animal models with AD, no drugs that focus on autophagic pathway have been demonstrated to be effective for clinical patients [11]. The reason may be related to (a) the duality of autophagy. The activation of autophagy triggers different signaling pathways, showing dual role. The net result depends on the comprehensive interaction between beneficial and harmful effects; (b) the activation of microglia. In microglia, autophagy activation has beneficial effects through the clearance of Aβ peptides, while the release of inflammatory cytokines may induce a detrimental neuron death; (c) limited effect. The independent application of autophagy-related drugs may be not enough to prevent the progression of advanced AD.

### Perplexity

Neuron survival is associated with autophagy signaling pathway. However, contradictory consequences have been observed following the modification of autophagy. For instance, IGF-I signaling is implicated in cellular senescence [119, 120], but neuronal IGF resistance prevents Aβ accumulation in the pathogenesis of AD [79, 121]. The neuronal IGF-1R ablation preserves autophagic compartment and enhances the systemic elimination of cytotoxic Aβ peptides. The blockage of IGF signaling in adult neurons can relieve the neuropathology of AD via Aβ clearance [35, 77]. Spatial memory in APP/PS1 mice is improved subsequent to the gene knockout of neuronal IGF-1R. Usually, IGF signaling promotes cell survival and proliferation, but the blockage of IGF pathway has profound effects on neuronal proteostasis and morphological maintenance via autophagic Aβ clearance.

### Novel strategy

The transplanted BMMSCs can differentiate into neurons, which plays a critical role in synaptogenesis and improvement of cognitive function [26, 122]. Therefore, the transplantation of BMMSCs is superior to drugs, especially in the advanced stage of AD with more neuronal loss. In addition, there is a low survival of transfused BMMSCs in the recipient, which is a real problem in the clinical application of stem cell therapy [13]. The activation of autophagy in BMMSCs may be a preconditioning and beneficial step before transplantation. It can be considered that transplanted BMMSCS is combined with autophagy-enhancing and/or anti-apoptotic drugs. Possibly, above effective combination is better than stem cell transplantation alone. A novel strategy involving BMMSCS plus drugs may be a new direction for the treatment of advanced AD.

## Conclusions

The present review provides the systematic and substantial coverage of autophagy mechanism by which the transplantation of BMMSCs improves cognitive and behavioral deficits in animal models with Alzheimer’s disease. The transplanted BMMSCs inhibit neuronal apoptosis and stimulate neurogenesis. The crosstalk between autophagy and apoptosis is a novel target for the development of therapeutic drugs. The therapeutic effect of stem cells may be enhanced by autophagy and/or apoptosis modulators.

## Data Availability

Not applicable.

## Abbreviations

AD: Alzheimer’s disease
BMMSCs: Bone marrow-derived mesenchymal stem cells
Aβ: Amyloid-beta
APP: Amyloid precursor protein
MSCs: Mesenchymal stem cells
iPS cells: Induced pluripotent stem cells
FGF: Fibroblast growth factor
CINC-1: Cytokine-induced neutrophil chemoattractant-1
IL-1β: Interleukin-1β
IL-6: Interleukin-6
IL-8: Interleukin-8
IL-10: Interleukin-10
TNF-α: Tumor necrosis factor-α
G-CSF: Granulocyte-colony stimulating factor
GM-CSF: Granulocyte-macrophage colony-stimulating factor
SCF: Stem cell factor
Seladin-1: Selective Alzheimer's disease indicator-1
VEGF: Vascular endothelial growth factor
NGF: Nerve growth factor
BDNF: Brain-derived neurotrophic factor
GSK-3: Glycogen synthase kinase-3
CXCL-12: C-X-C motif chemokine 12
CXCL-10: C-X-C-motif ligand 10
CCL5: Chemokine (C–C motif) ligand 5
Ang1: Angiopoietin 1
Ang2: Angiopoietin 2
Ang5: Angiopoietin 5
Ang7: Angiopoietin 7
LC3: Microtubule-associated proteins 1A/1B light chain 3B
LC3-II: Lipid modified form of LC3
Lamp-1: Lysosomal-associated membrane protein 1
mTOR: Mammalian target of rapamycin
HSPB1: Heat shock protein beta-1
Bcl-2: B-cell lymphoma 2
Bad: Bcl-2-associated agonist of cell death
Bcl-xL: B-cell lymphoma-extra
Bcl-w: Bcl-2-like protein 2
PI3K: Phosphoinositide 3-kinase
JNK: C-Jun N-terminal kinase
SDF-1: Stromal cell-derived factor 1
FGF: Fibroblast growth factor
FGF2: Fibroblast growth factor 2
BDNF: Brain-derived neurotrophic factor
PDGF: Platelet-derived growth factor
MIF: Macrophage migration inhibitory factor
IGF-1: Insulin-like growth factor 1
MCP-1: Monocyte chemoattractant protein 1
OPG: Osteoprotegerin
TGF-β: Transforming growth factor beta
TIMP-1: Tissue inhibitor of metalloproteinase 1
IAPs: Inhibitors of apoptosis proteins
Becn-1: Beclin-1
ERK1/2: Extracellular signal-regulated protein kinases 1 and 2
AMPK: 5' AMP-activated protein kinase
XIAP: X-linked inhibitor of apoptosis protein
C/EBPβ: CCAAT/enhancer-binding protein beta
Foxa2: Forkhead box protein A2
